# Auricle Pilomatricoma in Ear Lobule: A Case Report

**DOI:** 10.22038/IJORL.2023.66641.3281

**Published:** 2023-03

**Authors:** Mohammadreza Akhoundian yazd, Ermia Mousavi Mohammadi, Mohammadreza Afzalzade

**Affiliations:** 1 *Sinus and Surgical Endoscopic Research Center, School of Medicine, Mashhad University of Medical Sciences, Mashhad, Iran.*

**Keywords:** Ear lobule, Pilomatricoma

## Abstract

**Introduction:**

Skin tumors are prevalent in the head and neck especially auricle but pilomatricoma is extremely rare in ear lobule.

**Case Report:**

A 7-year-old girl without a history of previous illness presented with a 1.5-*month history* of a lesion in the *right ear lobule* that tended to grow in size. It was a 2*2*2cm *round, slightly painful lesion *with *soft* tissue and light red in color that yielded bloody or *serous fluid on aspiration*. The lesion was enucleated. The diagnosis was pilomatricoma.

**Conclusion:**

Although very rare, pilomatricoma should be considered as a differential diagnosis of ear lobule neoplasms.

## Introduction

Pilomatricoma is a differentiated *benign tumor derived from hair matrix* cells. This tumor accounts for 0.12% of all skin tumors (1, 2). Malherbe and Chenantais first described Pilomatricoma in 1880, and Malherbe coined the term calcifying epithelioma. Although the lesion was described as a benign tumor of the sebaceous glands, the perception of the morphological characteristics of the lesion has gradually increased since after. Later in 1961, Forbis and Helwig proved that the lesion originates from the cells of the hair matrix (3).

Pilomatricoma is often misdiagnosed and is not usually considered in differential diagnoses. *It usually appears* as a *superficial, firm, singular, slow-growing, painless mass in the skin. As the lesion forms, one may notice the covering skin discoloration or bluish tint (*3*). *

*In this case report, we present a young child with a mass diagnosed as pilomatricoma on the right earlobe, as an educational example, and a reminder to consider it in a differential diagnosis of ear lobule masses.*

## Case Report

**Fig 1:**
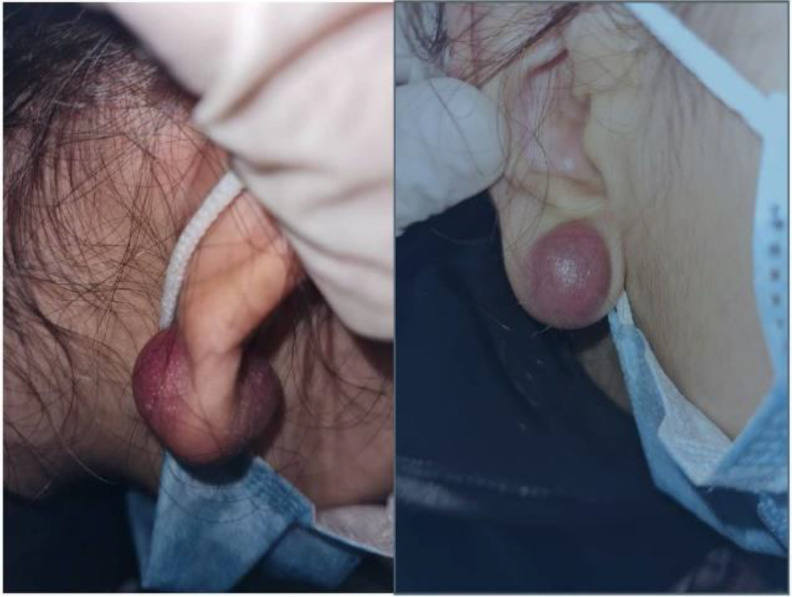
Image of the lesion arising in the atient's right ear lobule

**Fig 2:**
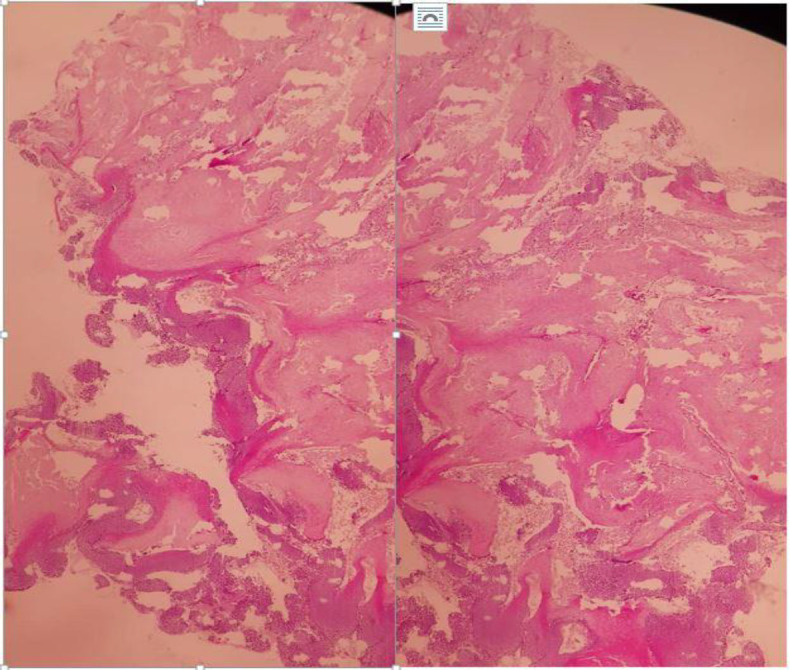
The sample pathology of the patient's right ear lesion showing islets of basaloid cells and islets of shadow cells

**Fig 3:**
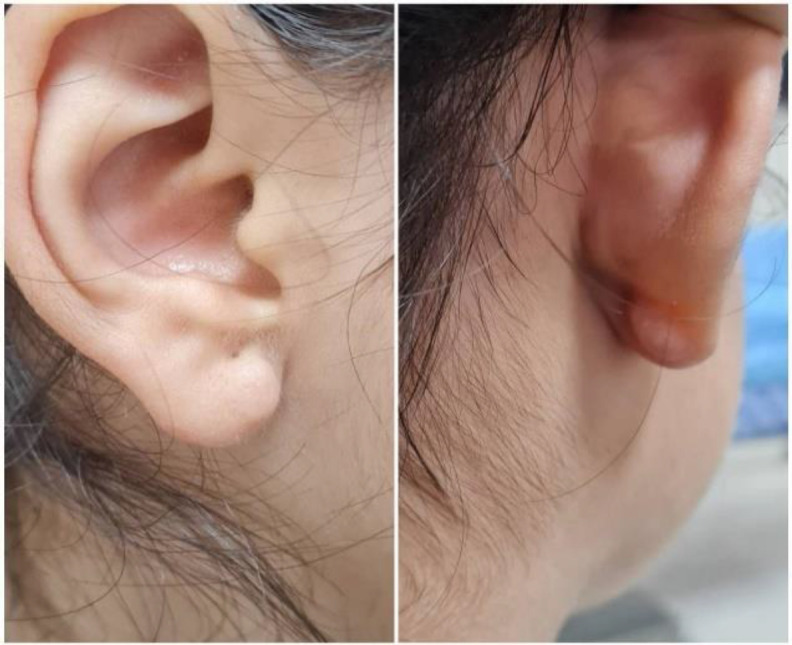
The ear lobule of patient 9 months after surgery

The patient was a 7-year-old girl without a history of previous illness, with a 1.5-*month history* of a growing lesion in the *right earlobe*. The patient had no prior history of trauma, pain, fever, chills, weight loss, fatigue, numbness, and a tingling sensation (pins-and-needles) on the lesion. The patient first visited a general practitioner and dermatologist and was treated with topical tetracycline ointment and cefixime syrup for a week, but did not respond to treatment and the lesion continued to increase in size. The patient then visited an otolaryngologist and underwent aspiration three times. The lesion yielded bloody or *serous fluid on aspiration*; however, it was still enlarging in size. With the possible diagnosis of colloid nodules, an intralesional injection was performed, after which the lesion growth was intensified. The patient came to our clinic with a 2*2*2cm sized *round, slightly painful lesion*, light red in color in the *right ear *lobule. Figure 1 shows the image of the lesion in the patient's *right earlobe*. The lesion was a soft tissue, nodule-like mass with slight tenderness on examination that yielded bloody or *serous fluid on aspiration*. The lesion was drained and enucleated. Pieces of tissue sections containing islets of basaloid cells and islets of shadow cells were observed in the biopsy specimen, indicating a pilomatricoma lesion. Figure 2 *shows the patient's sample pathology* report. After 9 months there were no recurrence (Figure 3)

## Discussion

Pilomatricoma occurs mostly in the head and neck area. This tumor is usually diagnosed in women in the first two decades of their lives (1). Pilomatricoma may be accompanied by some syndromes such as Turner (4). The tumor typically appears clinically as a rubbery subdermal mass. But , Usually the neoplasm diagnosed after resection during pathology exam because there are many lesion with similar characteristics, such as dermoid cyst, inflammatory lesion, lipoma, and hematoma (5). Pilomatricoma recurrence rates is ranging from 0% to 6% (6). These tumors have a 3: 2 prevalence in women, with head and neck and upper limb onset most often. The incidence of this neoplasm in healthy people does not investigated but in Turner syndrome studies demonstrated a high prevalence of pilomatricoma (2.6%) (6). 

*Histopathologic study of pilomatricoma* revealed different characteristic such as basaloid, polygonal squamous, shadow and foreign-body giant cells, and calcified deposits. All of these cells are rarely found in each patient , and therefore the exact cytological diagnosis of *pilomatricoma* is not easy (5). The lesions are often *misdiagnosed as epidermal inclusion cysts* when aspiration contains mainly squamous cells (6). The lesions are *misdiagnosed as* basal cell carcinoma if aspiration mainly shows basaloid cells. The lack of peripheral palisading in cell clusters and *nuclei* favors *pilomatricoma* (1). Smears showing mostly basaloid cells may easily be interpreted as small round cell tumors since they have a high nuclear ratio (7). 

Due to severe calcification in *pilomatricoma*, accurate diagnosis may be impossible (5). If aspiration mainly shows polygonal cells, the lesions may be misdiagnosed as metastatic squamous cell carcinoma. The lack of nuclear pleomorphism, large chromatin masses, atypical mitosis and detailed history allows us to make an accurate diagnosis (8). Imaging techniques are of little value in accurately diagnosing* pilomatricoma* (5). 

Definite diagnosis is typically made via cytologic study, showing basaloid and eosinophilic shadow cells arranged in lobules. The tumor shows the *predominant cell* population of *basaloid* in the early stages. With maturation of the neoplasm, the basophilic cells become shadow cells with no nuclei. These cells often calcify gradually , giving the lesion a firm consistency (3,5). 

Histopathologically, *pilomatricoma* should be distinguished from *basal cell carcinoma* with matricial differentiation (BCC-MD) and matricial carcinoma (9). The therapy* for pilomatricoma* is complete surgical excision (10). But enucleation as in our case also has been reported with no recurrence )^11^). Malignancy of the lesion is not common; however, it* should be suspected* in *cases* where *recurrence* is reported. 

## Conclusion

Although *pilomatricoma* is rare in auricle and lobule, it should be taken into account in differential diagnosis of lobule masses.
